# Nontypeable *Haemophilus influenzae* P5 Binds Human C4b-Binding Protein, Promoting Serum Resistance

**DOI:** 10.4049/jimmunol.2100105

**Published:** 2021-09-15

**Authors:** Oskar Thofte, Serena Bettoni, Yu-Ching Su, John Thegerström, Sandra Jonsson, Emma Mattsson, Linda Sandblad, Sara Martí, Junkal Garmendia, Anna M. Blom, Kristian Riesbeck

**Affiliations:** * Clinical Microbiology, Department of Translational Medicine, Faculty of Medicine, Lund University, Malmö, Sweden;; † Protein Chemistry, Department of Translational Medicine, Faculty of Medicine, Lund University, Malmö, Sweden;; ‡ Department of Molecular Biology, Umeå University, Umea, Sweden;; § Microbiology Department, Research Network for Respiratory Diseases, Bellvitge Institute for Biomedical Research, Bellvitge University Hospital, Barcelona, Spain; and; ¶ Instituto de Agrobiotecnología, Consejo Superior de Investigaciones Científicas–Gobierno de Navarra, Mutilva, Spain

## Abstract

Nontypeable *Haemophilus influenzae* (NTHi) is a Gram-negative human pathogen that causes infections mainly in the upper and lower respiratory tract. The bacterium is associated with bronchitis and exacerbations in patients suffering from chronic obstructive pulmonary disease and frequently causes acute otitis media in preschool children. We have previously demonstrated that the binding of C4b binding protein (C4BP) is important for NTHi complement evasion. In this study, we identified outer membrane protein 5 (P5) of NTHi as a novel ligand of C4BP. Importantly, we observed significantly lower C4BP binding and decreased serum resistance in P5-deficient NTHi mutants. Surface expression of recombinant P5 on *Escherichia coli* conferred C4BP binding and consequently increased serum resistance. Moreover, P5 expression was positively correlated with C4BP binding in a series of clinical isolates. We revealed higher levels of P5 surface expression and consequently more C4BP binding in isolates from the lower respiratory tract of chronic obstructive pulmonary disease patients and tonsil specimens compared with isolates from the upper respiratory tract and the bloodstream (invasive strains). Our results highlight P5 as an important protein for protecting NTHi against complement-mediated killing.

## Key Points

- Exposure of P5 at the surface of NTHi positively correlates with C4BP binding.
- C4BP bound to the bacterial surface retains its complement inhibitory capacity.
- C4BP binding to P5 is important for NTHi serum resistance.

## Introduction

Nontypeable *Haemophilus influenzae* (NTHi) is one of the main pathogens of the human respiratory tract (1). This Gram-negative bacterium is usually found as a commensal in preschool children and causes a variety of infections, the majority of which are confined to the respiratory mucosa. Together with *Streptococcus pneumoniae*, it is the most common cause of bacterial acute otitis media (AOM) and sinusitis (2–4). Furthermore, NTHi is a common pathogen found in patients with pneumonia or acute exacerbations of chronic obstructive pulmonary disease (COPD) and is frequently also seen in patients with cystic fibrosis (5–9). Because conjugated pneumococcal vaccines are now included in most child immunization programs worldwide, there is a concern regarding corresponding increased incidences of respiratory tract infections associated with NTHi (10, 11). Additionally, an increased incidence of invasive NTHi disease has also been observed after the introduction of the polysaccharide vaccine against *H. influenzae* capsule type b (12–16).

Successful colonization and infection of NTHi is dependent on surviving first encounters with the host innate immune system. This includes contact with the mammalian complement system, the first line of defense against pathogenic microorganisms (17). Complement resistance of NTHi has been linked to increased invasiveness and sepsis severity (18, 19). NTHi is likely to encounter complement proteins on inflamed mucosal surfaces in the lower airways during pneumonia or acute exacerbations of COPD and in middle ear exudate in AOM.

Complement activation leads to the deposition of C3b on the bacterial surface, with subsequent opsonization, phagocytosis, and bacterial lysis by formation of the membrane attack complex (MAC). The complement cascade can be activated by three different pathways: the classical, lectin, and alternative pathways. The classical pathway is activated by Abs (IgM and specific subclasses of IgG), whereas the alternative and lectin pathways are spontaneously activated by differences in bacterial membrane composition compared with the host cells. All three pathways lead to the formation of a C3-convertase, either the classical C3-convertase C4bC2a (classical and lectin pathway) or the alternative C3-convertase C3bBb (alternative pathway) that are crucial for C3b deposition on the cell surface and further downstream activation (20).

To avoid excessive activation of the immune system and subsequent host tissue damage, the complement system is strictly controlled by regulatory proteins that are utilized by NTHi (17, 21). C4b binding protein (C4BP) is the main soluble inhibitor of the classical and lectin pathways, whereas factor H (FH) is the main inhibitor of the alternative pathway. NTHi recruitment of vitronectin can also inhibit the terminal pathway (17, 21). C4BP, a large 500-kDa glycoprotein, consists of seven α-chains and one β-chain encompassing the 8 and 3 complement control protein (CCP) domains, respectively. C4BP inhibits the classical and lectin pathways by acting as a cofactor in factor I–mediated proteolysis of C4b and a decay-accelerating factor for the convertase. The effect is degradation of the classical C3-convertase C4bC2a and displacement of C2a from formed C3 convertases (22).

Surface recruitment of C4BP is a common strategy that has evolved in parallel between viruses, fungi, and a plethora of bacterial species. C4BP protects pathogens from opsonization, subsequently diminishes phagocytosis, and prevents MAC formation on the surface of Gram-negative bacteria (23). C4BP binding to *S. pneumoniae* is mediated by two surface proteins (PspA and PspC) that interact with CCP1–2 and CCP8 of C4BP (24, 25). In contrast, *Moraxella catarrhalis* ubiquitous surface protein (Usp) A1 and A2 bind CCP2 and CCP7 (26). Another example is *Neisseria gonorrhoeae* that binds CCP1–2 domains of C4BP through its major outer membrane protein (Omp) porin B (27). We have previously demonstrated that NTHi binds C4BP by interacting with CCP2 and CCP7, making the bacteria more resistant to complement-mediated killing (28). The NTHi ligand responsible for the binding of C4BP has, however, remained elusive.

In this study, we defined the NTHi Omp protein 5 (P5) as a ligand of C4BP. P5 is a member of the OmpA family with conserved transmembrane domains and four highly variable surface-exposed loops (29, 30). P5 has also been implicated in mediating binding to respiratory epithelial cells and mucin for adherence to host airway mucosa as well as interacting with FH, resulting in increased complement resistance (31–33). In this study, we show that P5 of NTHi mediates complement resistance by effectively binding human C4BP, which results in decreased complement deposition and increased bacterial survival. Finally, because bactericidal C4BP–IgM fusion protein was recently found effective in killing of *N. gonorrhoeae* (34) and *M. catarrhalis* (35), we also explored the effect of C4BP fusion proteins on NTHi serum resistance.

## Materials and Methods

### Bacterial strains and culture conditions

NTHi 3655 was a kind gift from Dr. R. Munson (The Ohio State University, Columbus, OH) (36). NTHi KR271 was a clinical isolate from our department (Table I). Clinical isolates from the upper respiratory tract (URT) were obtained from nasopharyngeal samples analyzed at the clinical microbiology laboratory at Skåne University Hospital (Lund/Malmö, Sweden). Blood and cerebrospinal fluid isolates used in this study were from a collection of invasive clinical isolates obtained in Sweden between 1997 and 2009 and previously described by Resman et al. (13, 37). Tonsil specimens were obtained from patients undergoing tonsillectomy with the indications of hypertrophy or recurrent acute tonsillitis (ethics approval number BD46/2007) at Skåne University Hospital (38). NTHi isolates from the lower respiratory tract (LRT) were obtained from sputum samples of COPD patients (Global Initiative for Chronic Obstructive Lung Disease stage II–IV, ages 61–84 y) analyzed at the microbiology department of Hospital Universitari Bellvitge (Barcelona, Spain) between 2010 and 2013. NTHi wild-type and mutant strains were routinely cultured in brain–heart infusion liquid broth supplemented with NAD and hemin (both at 2 μg/ml) or on chocolate agar plates at 35.5°C in a humid atmosphere containing 5% CO_2_. The P5-deficient mutants were cultured in the presence of 10 µg/ml chloramphenicol (Merck, Kenilworth, NJ). *Escherichia coli* BL21 (DE3) and DH5α were grown in Luria–Bertani (LB) liquid broth or on LB agar, whereby *E. coli* expressing recombinant P5 were cultured in the presence of 100 µg/ml ampicillin (Sigma-Aldrich, Saint Louis, MO). *N. gonorrhoeae* was cultured as previously described (34).

### Purification of human C4BP and preparation of C4BP fusion proteins

Human C4BP was purified from Na-citrate plasma isolated from blood collected from healthy volunteers using barium chloride precipitation to enrich the C4BP–protein S complex, followed by anion exchange chromatography and gel filtration (39). C4BP–IgM and C4BP–IgG fusion proteins were obtained from supernatants of stably transfected Chinese hamster ovary cells, as described recently (34). C4BP–IgM and C4BP-Fc were purified by affinity chromatography using a specific anti-C4BP Ab (MK104, produced in-house) (40) and a protein A column (GE Healthcare, Chicago, IL), respectively. Plasma-purified C4BP and C4BP fusion proteins were fluorescently labeled using Alexa Fluor 647 (A30009) and Alexa Fluor 488 (A10235) Microscale Protein Labeling Kits from Molecular Probes, respectively.

### P5 peptide synthesis and Ab production

A series of synthetic peptides corresponding to the four predicted outer surface loops (30) of P5 derived from NTHi strains 3655 and KR271 (Supplemental Table I) were synthesized by GenScript (Piscataway, NJ). Purified rabbit peptide polyclonal Abs (pAbs) directed against the outer surface loops 3 and 4 of P5 from NTHi 3655 (denoted as anti-P5_loop3^3655^ and anti-P5_loop4^3655^, respectively), and loop 3 of P5 from NTHi KR271 (anti-P5_loop3^KR271^) were purchased from GenScript.

### Two-dimensional SDS-PAGE and far–Western blot

Bacterial outer membrane fractions from an overnight culture were extracted and separated on a two-dimensional SDS-PAGE as previously described (41, 42). Briefly, 100 µg of bacterial proteins were first separated by isoelectric focusing on a precast 7-cm (pH 3–10) IPG gel strip (Immobiline Drystrips; GE Healthcare Biosciences) followed by a second dimension of gel electrophoresis on a 12% (v/v) SDS-polyacrylamide gel at 100 V for 90 min. Proteins on the gel were transferred onto a 0.45-µm Immobilon-PTM PVDF membrane (EMD Millipore, Bedford, MA) at 15 V for 16 h. Membranes were incubated with 100 µg of purified human C4BP (Complement Technology, Tyler, TX) in 2% PBS–BSA and detected with sheep anti-human C4BP pAbs (Abcam, Cambridge, U.K.) (1:1000 dilution) and HRP-conjugated donkey anti-sheep pAb (Abcam) (1:1000 dilution). Reactive Abs were detected with ECL Western Blotting Substrate (Pierce, Rockford, IL). Chemiluminescence signals on membranes were visualized on a ChemiDoc XRS+ System. C4BP binding protein spots were manually excised from the SDS-PAGE gel and analyzed by nano–liquid chromatography tandem mass spectrometry and MALDI-TOF (Alphalyse, Odense M, Denmark).

### Measurement of protein–protein interactions by biolayer interferometry

Kinetic analyses of the interaction between C4BP and outer membrane loops of P5 were performed by biolayer interferometry using a ForteBio Octet RED96 platform (Pall, Menlo Park, CA). Purified C4BP was immobilized on an amine-reactive (AR2G) sensor (Pall). The analyte (peptides corresponding to loops 1, 2, 3, and 4 of P5) were serially diluted in running buffer (PBS) ranging from 1.25 to 50 µM. The experiments were conducted at 30°C. Data analysis was performed using the ForteBio Data Analysis software 8.1 (Pall). Curves were fitted with 1:1 binding kinetics, and affinity (K_d_) was calculated.

### Generation of P5-deficient NTHi

Upstream (UF-P5) and downstream (DF-P5) flanking regions of the *ompP5* gene (GenBank [https://www.ncbi.nlm.nih.gov/genbank/] accession numbers EDJ92910 for NTHi 3655 and MW417498 for NTHi KR271) were first PCR amplified from bacterial genomic DNA with the primers listed in Supplemental Table I. The upstream (UF-P5) and downstream (DF-P5) flanking regions of the ompP5 gene from NTHi KR271 presented in this article have been submitted to GenBank (https://www.ncbi.nlm.nih.gov/genbank) under accession number MW417498. Open reading frame (ORF) of antibiotic chloramphenicol acetyltransferase gene (*cat*) (AY219687.1) was PCR amplified from pLysS plasmid (Novagen, Birmingham, U.K.). We thereafter performed overlapping PCR as described to generate a linear *P5-*knockout cassette carrying *cat* inserted between the UF-P5 and DF-P5 (41, 43). To knockout P5 expression from the genome of NTHi 3655 and KR271, competent NTHi were prepared and transformed with the *P5-*knockout cassette, as previously described (44). Transformants NTHi 3655Δ*ompP5* and NTHi KR271Δ*ompP5* were thereafter selected on chocolate agar containing chloramphenicol (10 µg/ml).

### Heterologous expression of NTHi P5 on the surface of *E. coli*

Full-length ORF of P5 were amplified from genomic DNA of NTHi 3655 and KR271 using primers containing specific restriction sites (Supplemental Table I). Amplicons were digested with FastDigest *Nco*I and *Nde*I (Thermo Fisher Scientific, Waltham, MA) for directional cloning into expression vector pET16b (Novagen) to yield recombinant P5^3655^-pET16b and recombinant P5^KR271^-pET16b carrying P5 ORF from NTHi 3655 and KR271, respectively. The recombinant plasmid constructs were subsequently transformed into *E. coli* DH5α for plasmid propagation. Transformants were selected on LB agar containing ampicillin (100 µg/ml). For P5 expression on the surface of *E. coli*, P5^3655^-pET16b and P5^KR271^-pET16b were transformed into *E. coli* BL21 (DE3) yielding strain *E. coli::ompP5^3655^* and *E. coli::ompP5^KR271^,* respectively, and induced with 1 mM isopropyl β-d-thiogalactoside, as described previously (41).

### SDS-PAGE and Western blotting

Whole-cell lysate of bacteria (1 × 10^7^ CFU) resuspended in PBS was heat denatured at 95°C for 10 min in SDS-reducing sample buffer (50 mM Tris–Cl [pH6.8], 2% SDS, 6% glycerol, 1% 2-ME, 0.004% bromophenol blue). Samples were separated on a 12% SDS-polyacrylamide gel at 150 V for 60 min and subsequently electrotransferred onto a 0.45-µm Immobilon-P PVDF membrane at 16 V for 15 h. Thereafter, membranes were blocked in PBS–0.05% Tween 20 (PBST) containing 5% skim milk. Membranes were incubated at room temperature for 1 h with rabbit anti-P5_Loop3^3655^or anti-P5_loop3^KR271^ diluted 1:1000 in 5 ml PBST–milk. Following three washes in PBST, membranes were incubated for 1 h with HRP-conjugated goat anti-rabbit pAbs (Abcam) diluted 1:1000. The membranes were finally washed in PBST, and signals were developed using ECL Western Blotting Substrate and visualized on a Bio-Rad Laboratories ChemiDoc, as described above.

### Flow cytometry

Bacteria were stained with 5 μM of CellTrace calcein violet (Thermo Fisher Scientific) and resuspended in HBSS containing 0.15 mM CaCl_2_ and 1 mM MgCl_2_ (HBSS^++^) (Life Technologies, Thermo Fisher Scientific). The binding of C4BP or C4BP fusion proteins to bacteria was measured by flow cytometry after incubation at 37°C for 30 min with the purified Alexa Fluor 647– or 488–labeled proteins (20 µg/ml for C4BP or 10 µg/ml for C4BP–IgM/C4BP–IgG), diluted in HBSS^++^, analyzed using a CytoFLEX flow cytometer (AW45306; Beckman Coulter, Brea, CA). P5 expression was measured using rabbit anti-P5_loop4^3655^ pAbs (1:500) and secondary FITC-conjugated goat anti-rabbit pAb (1:200) (Abcam) diluted in PBS plus 1% BSA. Of note, anti-P5_loop4^3655^ pAb can universally detect P5 derived from strains NTHi 3655 and KR271 at the bacterial cell surface when analyzed by flow cytometry. Bacteria were identified as calcein violet–positive events, and mean fluorescent intensity (MFI) was calculated in R 3.6.2 (45). Binding of C4BP and deposition of C3d from normal human serum (NHS) was detected with anti-human C4BP Ab MK67 (specific for CCP4 domain of the α-chain of C4BP) (40) and anti-human C3d pAb (Dako/Agilent Technologies, Santa Clara, CA), respectively. Bacteria were incubated with NHS (NTHi 3655: 3%, NTHi KR271: 5%, *E. coli*: 0.2%) diluted in HBSS^++^ for 30 min at 37°C, washed, and incubated with primary Abs (1:1000) on ice for 30 min. Finally, bacteria were labeled with secondary FITC-conjugated goat anti-rabbit pAb (Abcam) and analyzed using a FACSVerse flow cytometer (Becton Dickson, Franklin Lakes, NJ). Bacteria stained with a secondary Ab only, following NHS incubation, were considered as background, and the MFI ratio was calculated according to MFI as follows for each strain and experimental condition: (primary + secondary)/MFI (secondary).

### Scanning electron microscopy

For scanning electron microscopy, bacterial suspensions were fixed in 2.5% glutaraldehyde in 0.1 M sodium cacodylate overnight at 4°C. Bacteria were attached to poly-l-lysine–coated glass coverslips and dehydrated with an ascending ethanol series. The specimens were dried in a critical point dryer and sputtered with 2 nm platinum, and morphology was imaged by field-emission scanning electron microscopy (Carl Zeiss MERLIN; Carl Zeiss, Oberkochen, Germany) using a secondary electron detector at accelerating voltage of 5 kV and probe current of 120 pA.

### Transmission electron microscopy

For transmission electron microscopy (TEM), bacterial specimens were prepared in PELCO BioWave. Resuspended pellets were fixated in 2.5% glutaraldehyde in 0.1 M sodium cacodylate for 14 min. The samples were treated with 1% OsO_4_ for 14 min and rinsed twice with Milli-Q water. Specimens were further dehydrated in an ethanol gradient series and infiltrated with increasing concentrations of LR White resin in ethanol (1:3, 1:1, 3:1) and, finally, 100% resin. Samples were finally polymerized overnight at 65°C. Ultrathin 70-nm sections were obtained with an ultramicrotome (Leica Microsystems, Wetzlar, Germany) with a diamond knife (Diatome, Nidau, Switzerland), placed on formvar-coated copper grids and contrasted with uranyl acetate and lead citrate. The samples were examined with an FEI Talos L120 (Thermo Fisher Scientific, former FEI) TEM, and micrographs were acquired with an FEI Ceta CMOS (Thermo Fisher Scientific, former FEI) 16,000 pixel camera. Electron microscopy sample preparation and analyzes were performed at Umeå Centre for Electron Microscopy at Umeå University (Umeå, Sweden).

### Serum resistance

NHS was prepared from pooled blood obtained from healthy volunteers with informed consent. Heat-inactivated serum was prepared by inactivation at 56°C for 30 min. Optimal NHS concentrations were based on previous works (41, 43, 46, 47) and empirically determined in the current setting. Aliquots of bacteria (1 × 10^6^ CFU) were diluted in 25 mM veronal buffer (pH 7.3), 2.5% glucose, 1 mM MgCl_2_, 0.15 mM CaCl_2_, and 0.1% gelatin (DGVB^++^) and incubated in NHS (between 0.4–7.5%, depending on the strain tested, heat-inactivated serum or NHS plus Mg-EGTA (4 mM MgCl_2_, 10 mM EGTA) in a final volume of 700 µl at 37°C. At different time points, starting at T_0_ = 0 min, 100-µl aliquots were plated on chocolate agar (NTHi) or LB agar (*E. coli*) plates and incubated at 37°C. To study the effects of C4BP, bacterial samples were preincubated with C4BP (10 µg/ml) at room temperature for 30 min before dilution in DGVB^++^. Each experiment was performed in duplicate, and the percentage of survival was calculated as 100×(CFU at T_t_)/(CFU at T_0_). Because differences in growth rates can significantly affect serum resistance assays, all strains were also tested in heat-inactivated human serum in DGVB^++^ buffer (not shown). As expected, bacteria were not killed in the DGVB^++^ buffer only.

### Serum bactericidal assay with C4BP fusion proteins

Bacteria were cultured on chocolate agar plates overnight, subcultured in brain–heart infusion medium supplemented with 2 μg/ml both NAD and hemin. After 16 h, bacteria were subcultured in fresh medium for 4 h and thereafter washed once in PBS. Approximately 1 × 10^7^ CFU/ml harvested NTHi in gelatin veronal buffer with Mg and Ca (GVB^++^) were incubated for 30 min at 37°C with 20 or 50 μg/ml C4BP–IgG or C4BP–IgM, respectively. Thereafter, 5 or 3% of NHS diluted in GVB^++^ as a complement source was added to the wells containing NTHi KR271 or NTHi 3655, respectively, followed by incubation at 37°C. Aliquots of 25-μl reaction mixtures were collected at the initiation of the assay (t0) and after 30 min of incubation (t30). Samples were diluted in PBS and plated onto chocolate agar as three technical repetitions. Survival was calculated as a percentage between the number of viable CFU/ml at t30 relative to t0.

### Statistics

A Student *t* test, one-way or two-way ANOVA with Tukey post hoc test, or two-way repeated measures ANOVA with Bonferroni post hoc test was used for the analysis of parametric sets of data. Kruskal–Wallis nonparametric ANOVA with Dunn multiple pairwise comparisons were used for nonparametric data. Relational analysis was performed using Spearman rank correlation. Differences were considered statistically significant at *p* ≤ 0.05. All statistical analyses were performed in R 3.6.2 (45).

## Results

### Nontypeable *H. influenzae* binds human C4BP by interacting with Omp P5

**FIGURE 1.**
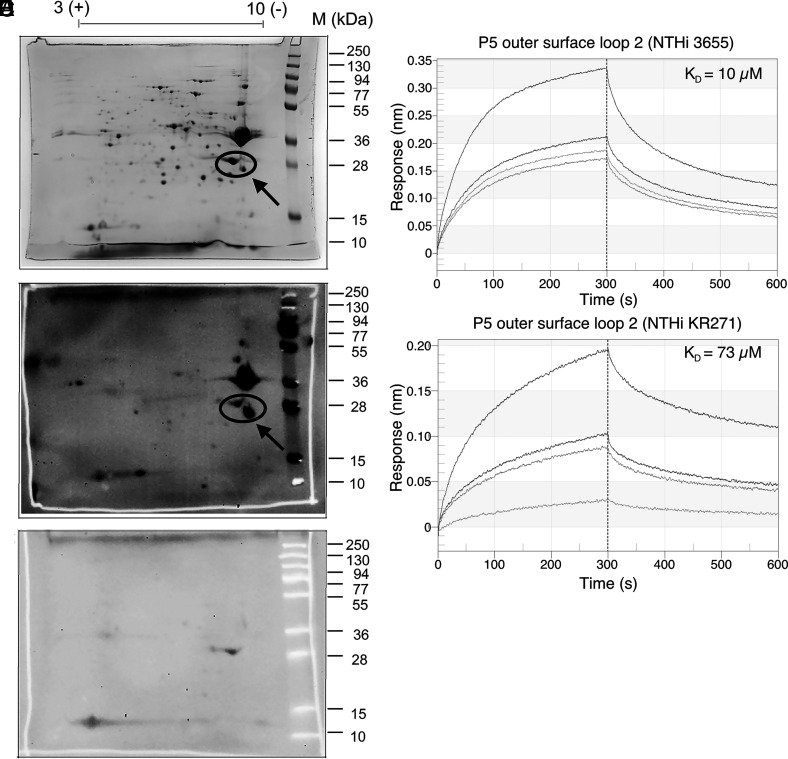
Identification of NTHi Omps interacting with human C4BP. Outer membrane fractions isolated from NTHi 3655 were subjected to two-dimensional SDS-PAGE. The gel was subsequently stained with Coomassie blue for visualization (**A**). A ligand overlay immunoblotting (far–Western blot) was performed by incubating the membrane with purified human C4BP, sheep anti-human C4BP pAb and HRP-conjugated donkey anti-sheep IgG pAb as a secondary layer (**B**). Incubation of a membrane without C4BP was also included as negative control to exclude unspecific signals (spots) caused by the Abs (**C**). The arrow shows Omp P5. The circle indicates multiple isoforms of P5. Binding kinetics of C4BP to P5 loop 2 from NTHi strains 3655 (**D**) and KR271 (**E**). The interaction was analyzed by biolayer interferometry (Octet RED96). Purified human C4BP was immobilized on AR2G sensors and the interaction with synthetic P5 peptides were analyzed at different concentrations (1.25–50 µM). Binding affinity (K_d_) was calculated by fitting the curves with 1:1 binding kinetics.

We have previously demonstrated that *H. influenzae* interacts with C4BP (28). To identify the Omp(s) responsible for the interaction, we performed a two-dimensional SDS-PAGE with the membrane fraction from NTHi 3655 (Table I) and probed with C4BP in a far–Western blot (Fig. 1A–C). C4BP binding protein spots were excised from the SDS-PAGE gel and analyzed by nano–liquid chromatography tandem mass spectrometry and nanoLC-MS/MS and MALDI-TOF. Proteomic analysis revealed the C4BP binding spot as NTHi 3655 Omp P5 with a Mascot score of 745 and 44% of sequence coverage.

**Table I.** List of strains used in this study

Bacterial Strain	Description/Genotype	Reference
NTHi 3655	Clinical isolate from a 10-y-old child with AOM	(36)
NTHi 3655Δ*ompP5*	Cm^R*a*^. NTHi 3655 with *ompP5* gene replaced by a chloramphenicol resistance gene	This study
NTHi KR271	Clinical isolate from blood culture of a 75-y-old individual with bacteremia	(37)
NTHi KR271Δ*ompP5*	Cm^R^. NTHi KR271 with *ompP5* gene replaced by a chloramphenicol resistance gene	This study
*E. coli* BL21(DE3)	F-*ompT hsdSB* (r_B_- m_B_-) *gal dcm* (DE3)	Novagen
*E. coli::ompP5* ^3655^	Amp^R*b*^. *E. coli* BL21 (DE3)–bearing recombinant plasmid of pET16b with *ompP5* ORF insertion expressing recombinant P5 from NTHi 3655 at the surface.	This study
*E. coli::ompP5* ^KR271^	Amp^R^. *E. coli* BL21 (DE3)–bearing recombinant plasmid of pET16b with *ompP5* ORF insertion expressing recombinant P5 from NTHi KR271 at the surface.	This study

a Cm^R^ is resistant to chloramphenicol.

b Am^R^ is resistant to ampicillin.

The clinical strain NTHi KR271 (Table I), isolated from a patient suffering from bacteremia, has previously been found to be more serum resistant compared with NTHi 3655 (data not shown). We, therefore, also included KR271 in the current study for further analysis in comparison with NTHi 3655. To define the C4BP-interacting region on P5 protein, we generated a series of synthetic peptides corresponding to the surface-exposed loops of P5 from NTHi strains 3655 (in this study denoted as P5^3655^) and KR271 (P5^KR271^) that were bioinformatically analyzed based upon previous work by Webb and Cripps (30). Eight predicted transmembrane spans and four outer surface loops have been defined in P5 of NTHi. Notably, pairwise sequence alignment between P5 from NTHi 3655 and KR271 revealed amino acid residue variations in all the predicted outer surface loops (Supplemental Fig. 1). Binding kinetics to human C4BP were therefore evaluated for all outer surface loops by biolayer interferometry. Purified human C4BP was immobilized on the sensor, and interactions with P5 loop peptides at increasing concentrations were analyzed. A dose-dependent binding was observed for outer surface loop 2 of both strains; the dissociation constant (K_D_) was calculated as 10 µM and 73 µM for surface loop 2 of P5^3655^ and P5^KR271^, respectively (Fig. 1D, 1E). This indicates that loop 2 might be the main binding site for human C4BP to both P5^3655^ and P5^KR271^.

### C4BP binding positively correlates with P5 expression in clinical NTHi isolates

**FIGURE 2.**
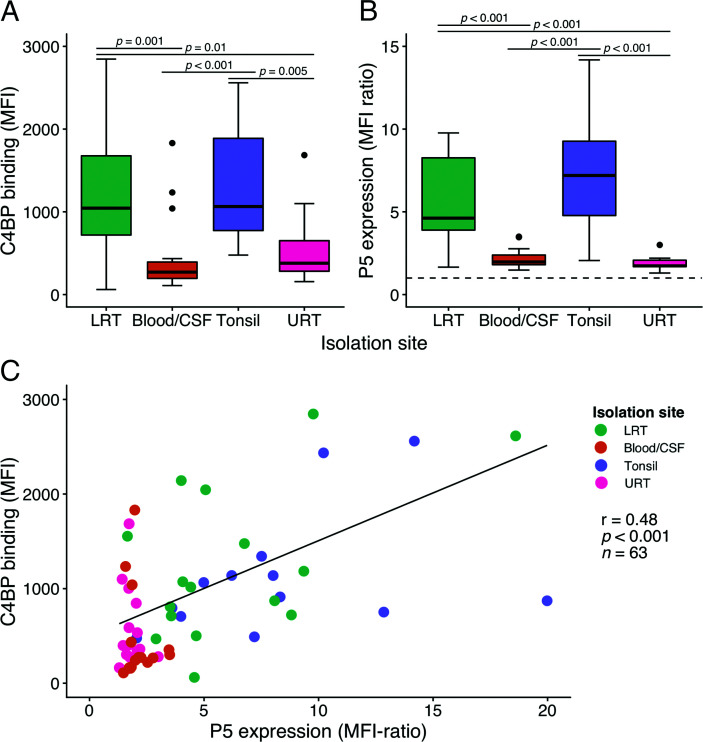
A positive correlation between NTHi P5 expression and C4BP binding is observed for clinical isolates. Box plots showing binding of Alexa Flour 647–labeled C4BP (20 µg/ml) to different NTHI isolates (**A**) and P5 surface expression assessed using an anti-P5 mAb and an FITC-conjugated secondary Ab (**B**) as measured by flow cytometry. NTHi strains were isolated from the LRT of COPD patients (LRT, *n* = 16), invasive isolates from blood or cerebrospinal fluid (Blood/cerebrospinal fluid, *n* = 16), tonsil specimens from patients undergoing tonsillectomy (Tonsil, *n* = 15) or the URT (*n* = 16). Vertical dashed line indicates cutoff for P5 expression. Statistical significance was calculated using Kruskal–Wallis nonparametric ANOVA. The lower, mid, and upper hinges mark the first quartile, the median, and the third quartile, respectively. Whiskers extend from the hinges to the lowest and highest value within 1.5 times the interquartile range. Outliers are plotted as dots. H(3) = 21.862, *p* < 0.001 and H(3) = 40.147, *p* < 0.001 with multiple pairwise comparisons according to Dunn. (**C**) Spearman correlation analysis of Alexa Flour 647–labeled C4BP binding versus P5 expression in NTHi clinical isolates. Data represent two separate measurements of technical duplicates. r = 0.48, *p* < 0.001, *n* = 63.

To determine the contribution of P5 expression levels to NTHi C4BP binding, we analyzed a series of NTHi strains (*n* = 63) isolated from different anatomical sites. The binding of purified human C4BP and P5 expression at the bacterial surface was measured by flow cytometry. We observed that clinical tonsil isolates of NTHi from patients undergoing tonsillectomy (38) had significantly higher C4BP binding compared with invasive isolates (*p* < 0.001) or isolates from the URT (*p* = 0.005; (Fig. 2A). In parallel, isolates from the LRT of COPD patients also showed higher binding levels in comparison with invasive (*p* = 0.001) and URT isolates (*p* = 0.01). Differences in P5 surface expression levels between groups showed a similar pattern as the C4BP binding capacity, with significantly (*p* < 0.001) higher expression in isolates from the LRT of COPD patients and tonsil specimens (Fig. 2B). Generally, large variations in both C4BP binding and P5 surface expression were seen within the groups and, in particular, NTHi isolated from tonsils or the LRT. Global analysis of the binding showed a significant positive correlation between C4BP binding and P5 surface expression (Spearman correlation analysis: *r* = 0.48, *p* < 0.001; (Fig. 2C).

### Decreased C4BP binding is observed for NTHi mutants devoid of P5

**FIGURE 3.**
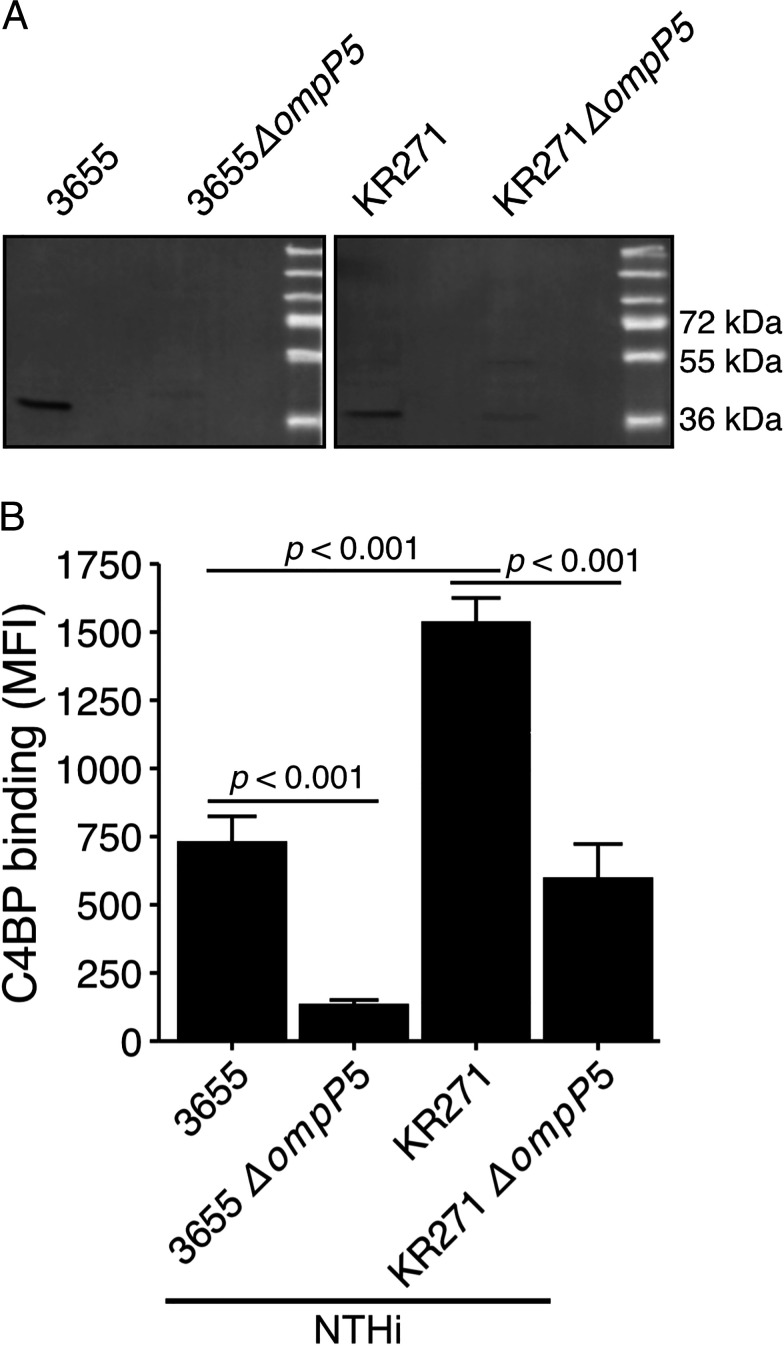
NTHi P5 mutants bind significantly less C4BP. (**A**) Evaluation of P5 expression in NTHi wild-type and P5-deletion mutants. P5 expression in wild-type and mutant strains detected by immunoblotting with rabbit anti-P5_loop3^3655^ and anti-P5_loop3^KR271^ Abs, respectively. (**B**) The NTHi strains 3655 and KR271, in addition to corresponding P5 mutant strains NTHi 3655Δ*ompP5* and KR271Δ*ompP5* were incubated with Alexa Flour 647–labeled C4BP (20 µg/ml), and binding was measured by flow cytometry. Each bar represents the mean ± SEM of five independent experiments. Statistical significance was calculated using one-way ANOVA. F(4, 10) = 42.23, *p* < 0.001, with Tukey post hoc test.

Because microbial interactions with host proteins can be mediated by multiple synchronous mechanisms, we aimed to determine the contribution of P5 to the interaction with human C4BP. We thus constructed NTHi *ompP5* deletion mutants (NTHi 3655Δ*ompP5* and NTHi KR271Δ*ompP5*; Table I). The absence of *ompP5* and P5 expression was confirmed by immunoblotting (Fig. 3A). Knockout of the P5 genes in NTHi 3655Δ*ompP5* and KR271Δ*ompP5* was also confirmed by PCR using P5 gene-specific primers. Amplicon of P5 gene was absent in the knockout mutants but present in their wild-type counterpart strains (data not shown). Both deletion mutants showed significantly (*p* < 0.001) decreased C4BP binding (Fig. 3B) compared with their respective wild-type counterparts, highlighting the importance of P5. We observed a significantly higher C4BP binding (*p* < 0.001) to NTHi strain KR271 compared with NTHi 3655. The knockout mutant NTHi KR271Δ*ompP5* also retained ∼40% of its C4BP-binding capacity, in contrast to NTHi 3655Δ*ompP5*, for which C4BP binding was almost completely abrogated. This may suggest additional C4BP-binding Omps in KR271 but not in NTHi 3655.

**FIGURE 4.**
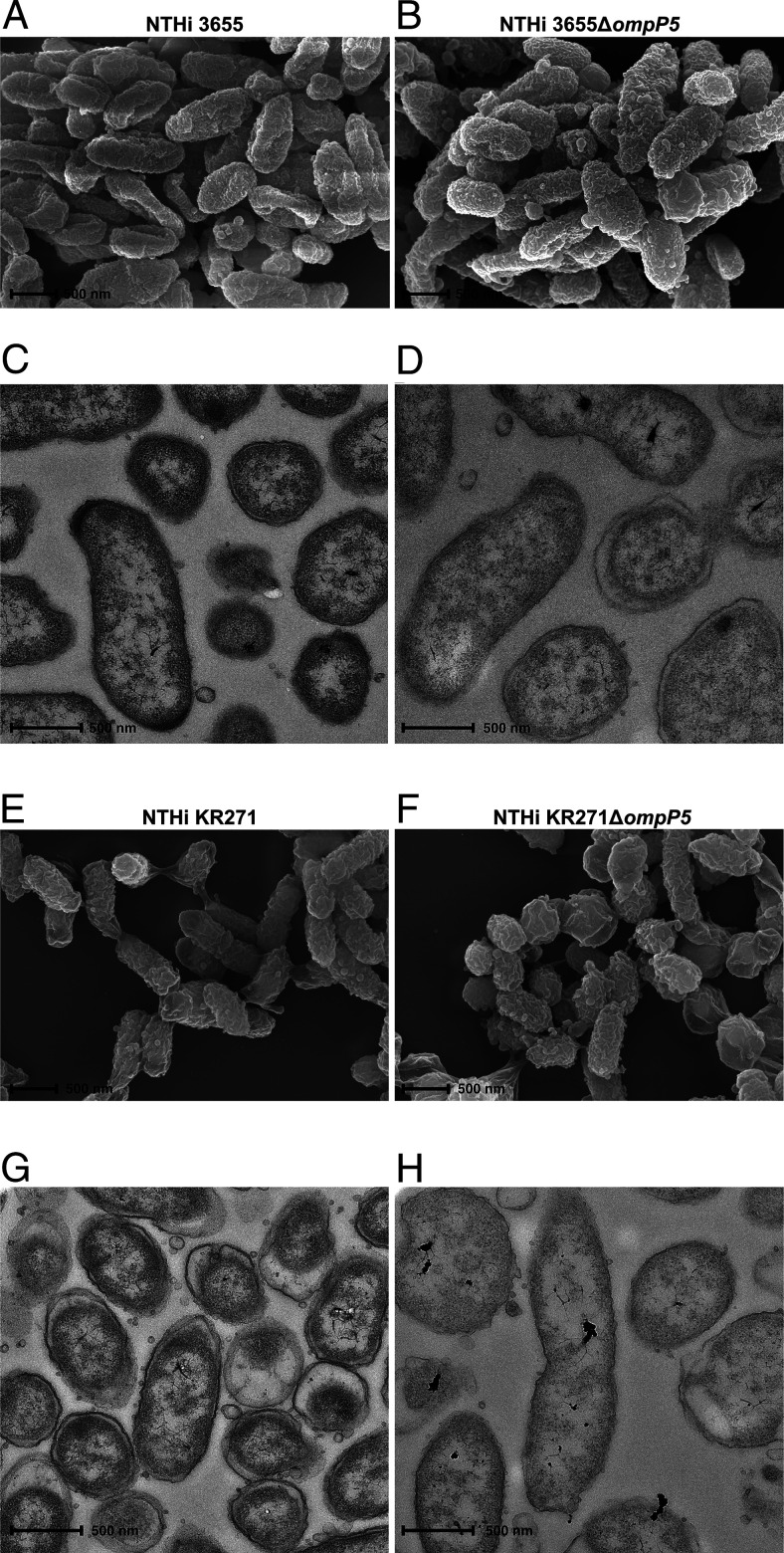
Scanning electron microscopy (**A**, **B**, **E**, and **F**) and TEM (**C**, **D**, **G**, and **H**) visualizing NTHi 3655 wild-type and NTHi 3655Δ*ompP5* (A–D) as well as NTHi KR271 wild-type and NTHi KR271Δ*ompP5* (E–H). Scale bar, 500 nm.

Whereas the crystal structure of P5 is currently unknown, in silico analysis has revealed that P5 shows sequence homology with OmpA of *E. coli* (48). Based on circular dichroism analysis, β-strand content in P5 is 49–55%, which is similar to OmpA (40% β-strand) (49). In *E. coli,* OmpA interacts with peptidoglycan in the presence of Braun lipoprotein to mediate cell wall attachment in *E. coli* (50). Considering the significant sequence similarity between P5 and OmpA, we sought to verify that the reduced C4BP binding in P5 mutants (Fig. 3B) was not merely a result of a disturbed cell wall. NTHi 3655, 3655Δ*ompP5* KR271, and KR271Δ*ompP5* were thus morphologically assessed by scanning electron microscopy and TEM (Fig. 4A–H). Importantly, intact cell walls and similar morphology could be seen between wild-type NTHi and mutant lacking P5 in their outer membrane layer.

**FIGURE 5.**
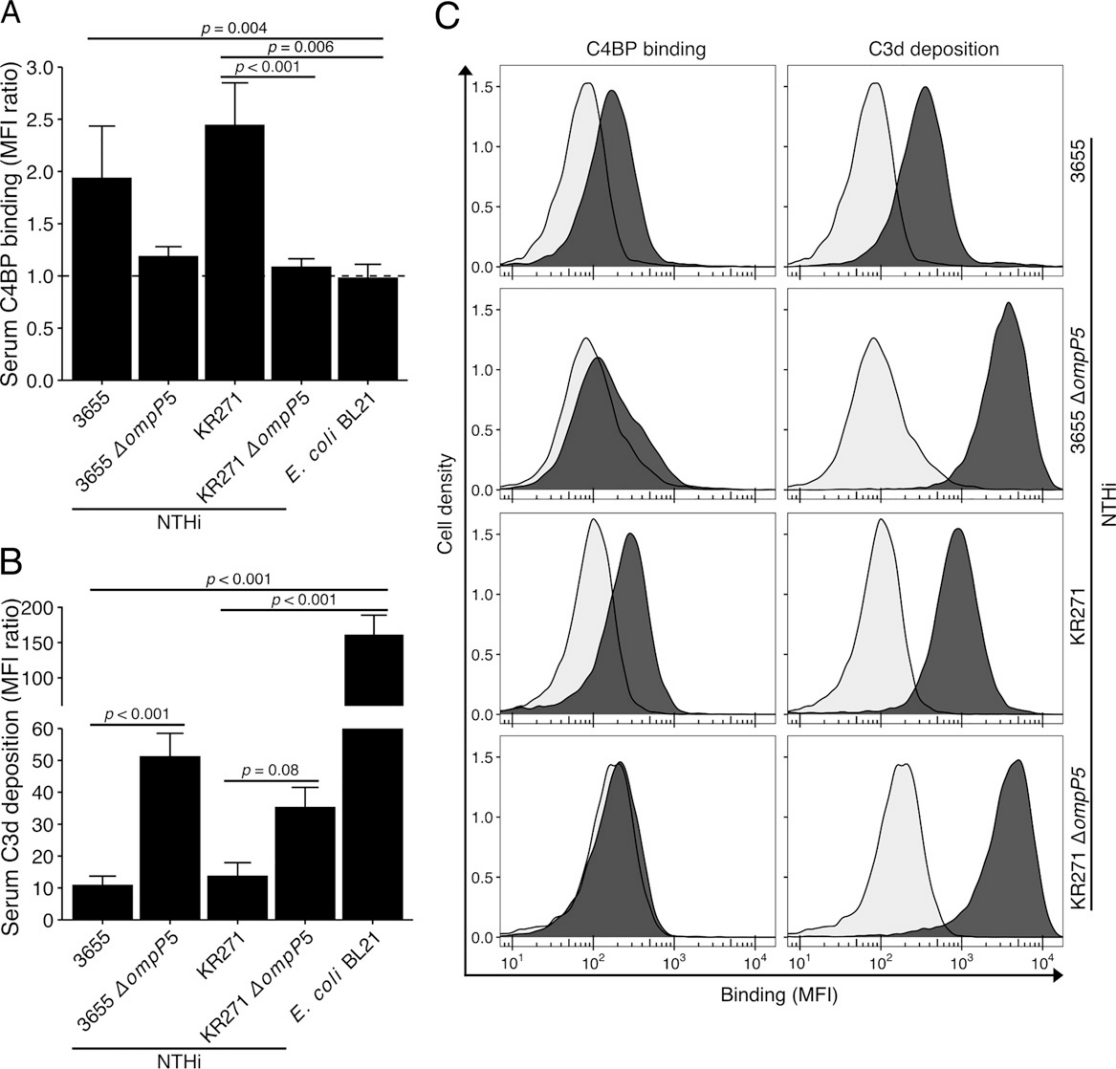
NTHi devoid of P5 binds significantly less human serum C4BP, resulting in more C3d deposition. NTHi strains 3655, KR271, and their corresponding P5 mutant strains NTHi 3655Δ*ompP5* and KR271Δ*ompP5* in addition to the laboratory strain *E. coli* BL21 were incubated with human serum (NHS: 3% and 5% for NTHi 3655 and KR271, respectively, and 0.2% for *E.coli* BL21) for 30 min at 37°C followed by flow cytometry analyses. (**A** and **B**) C4BP binding (A) and C3d complement deposition (B) on the bacterial surface was detected by specific Abs. Each bar represents the mean MFI ratio ± SEM of four and six independent experiments, respectively. MFI ratio was calculated as follows: MFI of cells incubated with primary and FITC-conjugated secondary Ab/MFI of cells incubated with secondary Ab only. Statistical significance was calculated using one-way ANOVA. (A) F(4, 15) = 6.54, *p* = 0.003; (B) F(4, 24) = 11.67, *p* < 0.001 with Tukey post hoc test. (**C**) Representative flow cytometry profiles from experiments included in (A) and (B). Background (light gray) represents bacteria incubated with FITC-conjugated secondary Abs only.

The P5 knockout mutants were next assessed for their ability to recruit C4BP from NHS. Bacteria were incubated with NHS from healthy donors, followed by flow cytometry analysis of C4BP binding and complement component C3d deposition at the bacterial surface. Both wild-type strains NTHi 3655 and KR271 readily bound C4BP derived from NHS (Fig. 5A, 5C). Interestingly, under these conditions, C4BP binding to the P5 deletion mutants were reduced for both mutant strains (NTHi 3655Δ*ompP5* and KR271Δ*ompP5*) compared with their parental wild-type strains. Concurrent measurement of surface C3d deposition revealed 5- and 4-fold increases for NTHi 3655Δ*ompP5* and KR271Δ*ompP5*, respectively, when compared with wild-type counterparts (Fig. 5B, 5C). Our findings indicate that the NTHi surface-bound C4BP maintains its inhibitory capacity.

### NTHi P5-dependent C4BP binding is important for resistance against the bactericidal activity of human serum

**FIGURE 6.**
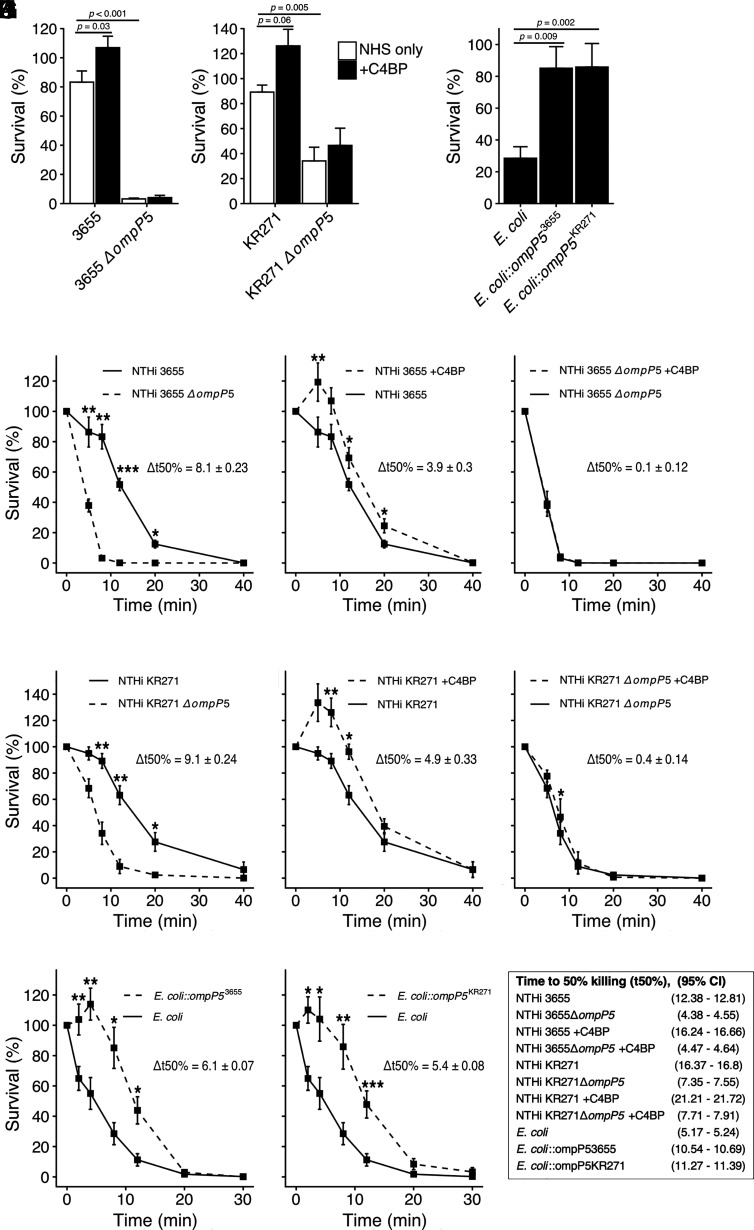
NTHi P5 is important for serum resistance. NTHi strains 3655, NTHi KR271, and corresponding P5 mutant strains NTHi 3655Δ*ompP5* and KR271Δ*ompP5* were incubated with strain-optimized concentrations of NHS (3655, 2% and KR271, 7.5%) at 37°C, and percentage survival was assessed at different time points. (**A** and **B**) Serum resistance of strains NTHi 3655 and NTHi 3655Δ*ompP5*, NTHi KR271, and NTHi KR271Δ*ompP5* after 8 min of incubation in NHS with or without preincubation with 10 µg/ml recombinant C4BP. (**C**) Serum resistance of naive *E. coli* BL21(DE3), *E. coli::ompP5*^3655^, and *E. coli::ompP5*^KR271^ after 8 min of incubation in NHS. (**D**) Serum resistance of wild-type strain NTHi 3655 and NTHi 3655Δ*ompP5* at multiple time points. (**E** and **F**) Serum resistance after preincubation of NTHi 3655 (E) or NTHi 3655Δ*ompP5* (F) with 10 µg/ml recombinant C4BP. (**G**) Serum resistance of wild-type strain NTHi KR271 and NTHi KR271Δ*ompP5*. (**H** and **I**) Serum resistance after preincubation of NTHi KR271 (H) or NTHi KR271Δ*ompP5* (I) with 10 µg/ml recombinant C4BP. (**J** and **K**) Serum resistance of naive *E. coli* BL21(DE3), *E. coli::ompP5*^3655^ (J), and *E. coli::ompP5*^KR271^ (K) incubated with 0.5% NHS at 37°C and assessed at different time points. Each bar represents the mean ± SEM of three to eight independent experiments with technical duplicates. Statistical significance was calculated using (A and B) two-way ANOVA. (A) F(1, 29) = 3.77, *p* = 0.0621, η^2^ = 0.01; strain effect: F(1, 29) = 243.8, *p* < 0.001, η^2^ = 0.82. (B) F(1, 26) = 1.28, *p* = 0.2686, η^2^ = 0.01; strain effect: F(1, 26) = 38.37, *p* < 0.001, η^2^ = 0.55. Significant main effects were followed by Tukey multiple comparisons test, (C) one-way-ANOVA, F(2, 24) = 9.22, *p* = 0.001, η^2^ = 0.43 with Bonferroni post hoc test, (D–K) repeated measures two-way ANOVA, (D) F(5, 20) = 50.71, *p* < 0.001, η^2^ = 0.64; strain effect: F(1, 5) = 57.23, *p* = 0.002, η^2^ = 0.67. (E) F(5, 20) = 10.80, *p* < 0.001, η^2^ = 0.14; C4BP effect: F(1, 5) = 24.28, *p* = 0.008, η^2^ = 0.20. (F) F(5, 20) = 0.05, *p* = 0.99, η^2^ = 0.002; C4BP effect: F(1, 5) = 0.07, *p* = 0.80, η^2^ < 0.001. (G) F(5, 25) = 5.79, *p* = 0.006, η^2^ = 0.25; strain effect: F(1, 5) = 31.60, *p* = 0.002, η^2^ = 0.40. (H) F(5, 25) = 8.77, *p* < 0.035, η^2^ = 0.12; C4BP effect: F(1, 5) = 7.25, *p* = 0.031, η^2^ = 0.17. (I) F(5, 25) = 2.49, *p* = 0.15, η^2^ = 0.03; C4BP effect: F(1, 5) = 5.46, *p* = 0.67, η^2^ = 0.02). (J) F(6, 24) = 18.09, *p* < 0.001, η^2^ = 0.39; C4BP effect: F(1, 8) = 5.46, *p* = 0.005, η^2^ = 0.41). (K) F(6, 24) = 14.70, *p* = 0.008, η^2^ = 0.22; C4BP effect: F(1, 8) = 20.60, *p* = 0.002, η^2^ = 0.30. t_50%_ was calculated from linear regression analysis of time (min) and proportion of dead bacteria. This was followed by analysis of simple main effect for strain or C4BP preincubation; statistical significance received Bonferroni adjustment, *p < 0.05, **p < 0.01, and ***p < 0.001 as indicated.

The role of Omp P5 for survival in human serum was explored by conducting serum resistance assays. NTHi 3655 and KR271 wild-type and their corresponding P5 mutant strains were incubated with NHS followed by an analysis of bacterial survival at different time points. Both mutants showed significantly lower serum resistance across multiple time points (*p* < 0.001 for NTHi 3655Δ*ompP5*; *p* = 0.005 for KR271Δ*ompP5*) (Fig. 6A, 6B, 6D, 6G), indicating the importance of P5-dependent complement inhibition. Interestingly, the time to 50% killing (t_50%_) was almost 3-fold longer for NTHi 3655 wild-type compared with NTHi 3655Δ*ompP5* (t_50%_ 95% CI [minutes], NTHi 3655: 12.38–12.81; NTHi 3655Δ*ompP5*: 4.38–4.55). For NTHi KR271, there was a 2-fold decrease seen for the P5 knockout strain compared with the wild-type (t_50%_ 95% CI [minutes], NTHi KR271: 16.37–16.66; NTHi KR271Δ*ompP5*: 7.35–7.91). To test for the effect of C4BP binding, all strains were preincubated with purified C4BP (10 µg/ml) prior to incubation with NHS. For wild-type strains NTHi 3655 and KR271, preincubation with C4BP led to significantly increased survival (Δt_50%_ 95% CI [minutes], NTHi 3655: 3.60–4.20; NTHi KR271: 4.57–5.23) (Fig. 6A, 6B, 6E, 6H). However, preincubation with C4BP had no relevant effect on serum resistance of the mutant strains NTHi 3655Δ*ompP5* or KR271Δ*ompP5* (Fig. 6A, 6B, 6F, 6I), which was in line with their reduced ability to bind C4BP.

To analyze whether the alternative pathway of complement activation was also involved in the total serum killing of wild-type NTHi and mutants as observed in (Fig. 6A and 6B, bacteria were incubated with NHS in the presence of Mg-EGTA. In this setting, both the classical and the lectin complement pathways are blocked (51). Such treatment did not show any retained bacterial killing activity (Supplemental Fig. 2), indicating that the alternative pathway was not involved at these NHS concentrations.

Next, we wanted to define the sole effect of P5 expression in the absence of the potential background of other Omps, in bacterial interaction with complement regulators. Thus, we transformed the serum-sensitive laboratory strain *E. coli* BL21(DE3) with *ompP5* derived from NTHi 3655 and KR271, respectively (Table I). When exposed to 0.5% NHS, *E. coli* expressing recombinant P5^3655^ (Fig. 6C, 6J) or recombinant P5^KR271^ (Fig. 6C, 6K) survived significantly (*p* = 0.002; *p* = 0.009) better than the naive *E. coli* BL21(DE3). The t_50%_ was doubled in both transformed strains (t_50%_ 95% CI [minutes], *E. coli*: 5.17–5.24; *E. coli::ompP5^3655^*: 10.54–10.69; *E. coli::ompP5^KR271^*: 11.27–11.39) These experiments highlight that P5 from NTHi is able to increase serum resistance in a heterologous expression host. Taken together, our results indicate that C4BP contributes to P5-dependent NTHi serum resistance.

### C4BP–IgM and C4BP–IgG fusion proteins bind to NTHi in a P5-dependent manner but do not promote bacterial killing

**FIGURE 7.**
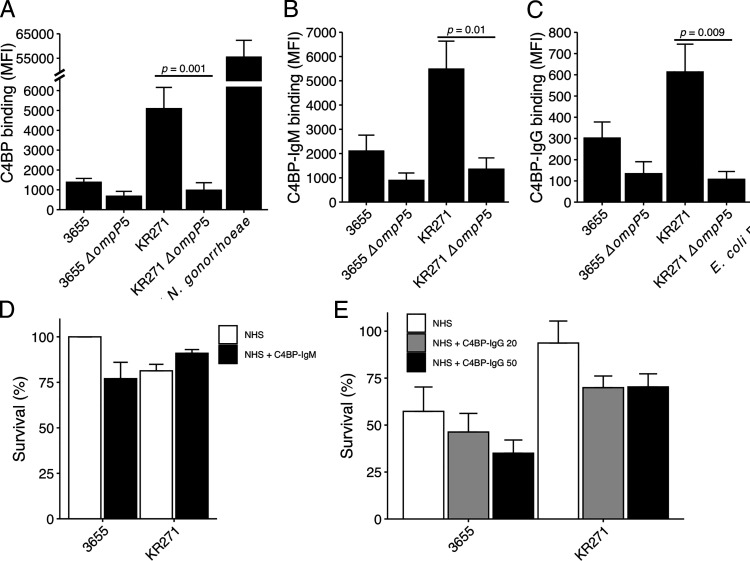
C4BP fusion proteins bind to NTHi but do not promote serum killing. (**A**–**C**) NTHi strains 3655 and KR271 and corresponding P5 mutant strains NTHi 3655Δ*ompP5* and KR271Δ*ompP5* were incubated with 10 μg/ml of fluorescently labeled C4BP (A), C4BP-IgM (B), or C4BP–IgG (C) for 30 min at 37°C, and binding was evaluated by flow cytometry. *N. gonorrhoeae* FA1090 strain was used as positive control for binding to C4BP. (**D** and **E**) Wild-type NTHi 3655 and KR271 were preincubated with or without C4BP–IgM (50 μg/ml) (D) or C4BP–IgG (20 or 50 μg/ml) (E) for 30 min at 37°C prior to addition of 3% and 5% NHS, respectively. Bacteria were counted at t = 0 and t = 30 min postaddition of serum, and survival percentage was calculated. Each bar represents the mean ± SEM of at least three independent experiments with technical duplicates. Statistical significance was calculated using one-way ANOVA. (A) F(3,14) = 8.955, *p* = 0.001; (B) F(3, 8) = 8.959, *p* = 0.006; (C) F(3,8) = 8.416, *p* = 0.007 with Tukey post hoc test.

C4BP fusion proteins are promising for adjunct therapy with conventional antimicrobials against bacterial pathogens (34, 35). By fusing C4BP α-chains to the Fc domain of IgG or IgM, the bacterial C4BP binding can be exploited to promote complement activation and MAC formation. This is mediated by the initiation of the classical pathway by the Fc portion deposited at the bacterial surface. Our C4BP–IgM is composed of C4BP CCP1 and CCP2 fused to IgM CH2-CH4, which assembles into a pentamer or hexamer upon expression (34). In contrast, the C4BP–IgG contains CCP1–2 and CCP4–8 (C4BPΔCCP3) fused to IgG Fc. Using flow cytometry, we measured the binding of both fusion proteins to the surface of NTHi 3655 and KR271 (Fig. 7A–C). For both wild-type strains, we observed a binding capacity similar to that of full-length C4BP with diminished binding of the fusion proteins in mutants devoid of P5. However, C4BP binding levels to NTHi were found to be 10-fold less than binding to the Gram-negative pathogen *N. gonorrhoeae* that was included for comparison as a positive control (Fig. 7A). *N. gonorrhoeae* has been shown to very efficiently bind C4BP (34). To determine whether C4BP–IgM and C4BP–IgG can enhance NTHi complement-mediated killing, we conducted serum resistance assays using human serum. C4BP–IgM did not promote decreased serum resistance under the current experimental conditions (Fig. 7D). In contrast, preincubation of NTHi with C4BP–IgG rendered a subtle dose-dependent killing that was not, however, statistically significant (Fig. 7E).

## Discussion

In this study we identified P5 to be important for the ability of NTHi to recruit the human complement inhibitor C4BP to the bacterial surface. The binding correlated with surface expression levels of P5 in clinical isolates, and mutants devoid of P5 showed significantly decreased C4BP binding. Furthermore, serum resistance assays revealed that the addition of C4BP increased survival in wild-type but not P5 mutant strains. Expression of NTHi P5 on the surface of serum-sensitive *E. coli* also resulted in increased survival, dependent on interference with the classical or lectin pathways of complement activation. Collectively, our data strongly suggest that P5 is important for C4BP-mediated complement evasion by NTHi.

Complement is an essential part of innate immunity, and complement resistance is crucial for bacterial colonization and survival. This is highlighted by the multitude of evasion strategies that have been evolved independently by different bacterial species (52). Interestingly, *H. influenzae* has developed several parallel countermeasures to avoid complement-mediated killing. We have previously demonstrated that *H. influenzae* readily binds C4BP, FH, and vitronectin to its surface, mediating resistance to the classical and lectin pathways (C4BP), the alternative pathway (FH), and the terminal cytolytic pathway (vitronectin) (28, 46, 53). Some of the mechanisms by which NTHi binds these inhibitors have been studied, and several bacterial Omps, including lipoprotein P4, protein E, and protein F have been shown to bind to vitronectin (41, 43, 46). In this study, we revealed that the Omp P5 of NTHi is the binding partner for C4BP on the bacterial surface. Interestingly, P5 also interacts with FH (54–56), likely because both complement inhibitor proteins possess CCP domains, which share common repeats important for the direct interaction with components of the complement cascades. Such bifunctionality is also seen in *S. pneumoniae*, in which PspC interacts with both FH and C4BP (25, 57). In this study, we showed that P5 knockout strains bind significantly less C4BP. It is nevertheless noteworthy that NTHi KR271Δ*ompP5*, which does not express P5, still exhibited residual binding to C4BP, suggesting the presence of other potential C4BP binding proteins in this strain but not NTHi 3655. The effect of P5 knockout on serum resistance in the NTHi KR271 strain (Fig. 6B; effect size [η^2^] = 0.55) was also slightly less dramatic compared with NTHi 3655 (Fig. 6A; effect size [η^2^] = 0.82). However, the effect on t_50%_ (Δt50%) of the P5-knockout (Fig. 6D, 6G) in addition to C4BP preincubation (Fig. 6E, 6H) was comparable in both strains, indicating that P5 is the most important C4BP binding partner of NTHi. Furthermore, the effect of C4BP preincubation in NTHi KR271Δ*ompP5*, albeit statistically significant (Δt50% = 0.4 ± 0.14), was less than 10% of the effect in wild-type NTHi KR271. When transformed into serum-sensitive *E. coli* BL21, P5 from NTHi 3655 or KR271 had similar effects on survival and t_50%_ (Fig. 6C, 6J, 6K), indicating that the difference in serum resistance and C4BP binding between the two strains is not due to differences in the P5 sequence but rather by P5 expression levels or interactions with other surface proteins. In both strains, P5 expression on the bacterial surface seems crucial for serum resistance based upon the fact that the NTHi P5 knockout NTHi KR271 strain had a halved t_50%_ (Fig. 6G) and ∼65% in NTHi 3655 (Fig. 6D). Strain variations cannot, however, be excluded because we only analyzed two strains in detail.

In a previous study, we observed no differences in binding of complement inhibitors (i.e., C4BP, FH, and vitronectin) between blood-derived and nasopharyngeal NTHi isolates (18). This indicates that complement resistance is equally important for NTHi strains regardless of the site of infection. The comparable levels of P5-expression and C4BP binding between nasopharyngeal and invasive isolates seen in the current study support this hypothesis (Fig. 2A, 2B). In this study, we also included clinical strains isolated from tonsils of patients suffering from chronic tonsillar infections or hypertrophy and from the LRT of patients suffering from COPD. These anatomical sites are classically involved in prolonged colonization (38, 58). Interestingly, these clinical isolates showed higher P5 expression levels and C4BP binding, indicating a different immunologic pressure at these colonization sites. Importantly, P5 is also known to be involved in adhesion and entry into both epithelial and phagocytic cells, functions that could also be more important for colonization of the tonsils or the LRT (31). Furthermore, C4BP binding also seems to promote endothelial cell adhesion and invasion (59) and plasminogen binding (60), in addition to inhibiting complement. These alternative functions of both P5 and C4BP could prove more important in the immune cell–rich tonsillar tissue or in the LRT, explaining the somewhat counterintuitive lower C4BP binding seen in the invasive isolates. The higher serum resistance seen in LRT strains is also in good agreement with previous work by Nakamura et al. (61), which compared LRT isolates from COPD patients with strains isolated from the URT of healthy children. Of note, all clinical isolates analyzed in the current study expressed P5 on their surface and were able to bind C4BP (Fig. 2A, 2B). Tonsil and LRT isolates, however, showed large variations in both P5 expression and C4BP binding. One possible explanation could be that the isolates are sampled at different stages of host-specific adaptation. During the colonization of pulmonary tissue, NTHi undergoes genetic adaptations involving lipooligosaccharide synthesis, iron uptake, immune evasion, and attachment to the host tissue (9). Recently, Pettigrew et al. (58) found that a single NTHi isolate can persist in individuals with COPD for months up to several years. The persistence duration was found to correlate with the number of altered simple sequence repeats. This suggests that NTHi specimens isolated at different stages of adaptation will differ variably from strains isolated from the human URT, the main reservoir of *H. influenzae.* In the same study, P5 was found to be highly diverse between strains and underwent changes under the selective pressure during persistence in outer surface loops 2 and 3 (58). NTHi colonization in tonsils is less studied, but a significant presence of NTHi in tonsil specimens after tonsillectomy has previously been reported by us and others (38, 62). Intracellular survival of NTHi within the tonsillar core has also been linked with resistance to antibiotic treatment, potentially promoting recurrent infection or chronic colonization (63). Further studies of the similarities between NTHi isolates from the tonsils and LRT are warranted. Especially interesting is the notion that tonsil colonization could provide a reservoir, enabling persistent or recurrent LRT infections in COPD patients, given that tonsil colonization requires similar adaptations.

In previous studies we showed that C4BP and FH fusion proteins of bacterial binding domains fused with constant portions of Ig could efficiently aid in eliminating bacteria through complement activation (34, 64–66). In particular, FH–Fc fusion molecules were shown to be effective in mediating complement-dependent lysis of NTHi. Encouraged by these findings, we explored, in this study, the ability of C4BP–IgM and C4BP–IgG chimeras in inducing complement-mediated killing of NTHi. We speculate that the lack of bactericidal activity of C4BP fusion proteins on NTHi (Fig. 7D, 7E) is due to lower affinity binding of the chimeras. *N. gonorrhoeae* FA1090, which is effectively killed by C4BP fusion proteins (34), showed up to 10-fold higher binding levels compared with NTHi KR271, indicating a far higher affinity for C4BP (Fig. 7A). Being hexameric, C4BP–IgM can effectively compete with heptameric full-length C4BP and trigger complement activation via the IgM moiety. However, it includes only CCP1 and CCP2 from the α-chain of C4BP and not the CCP7 domain involved in the interaction with NTHi (28). In contrast, C4BP–IgG contains all CCP domains of α-chain C4BP with the exception of the complement inhibitory domain CCP3. However, its dimeric form is not as potent in displacing native C4BP and promoting efficient complement-mediated cell lysis, as seen with *N. gonorrhoeae* (34). With the aim to develop an effective C4BP fusion protein targeting NTHi, some modifications to the C4BP–IgG fusion protein tested in this study are needed. By inserting the multimerizing mutations into the IgG moiety, which results in hexameric molecules, the protein would be better capable of competing with native C4BP (67).

In conclusion, we have explored the importance of P5 from NTHi for the interaction with human C4BP, with an emphasis on serum resistance. NTHi expresses a large repertoire of adhesive proteins, including P5, which contribute to its virulence. The binding of human C4BP has been shown to enhance adhesion and invasion into epithelial cells by various bacteria (59). In this study, we found that NTHi strains isolated from the LRT of COPD patients and tonsil specimens express more P5, which, in turn, positively correlates with C4BP binding. If these adaptations are driven by complement-mediated immunological pressure at these sites and/or alternatives, functions of both P5 and C4BP should be further investigated. C4BP recruitment to the surface of NTHi is nonetheless important for serum resistance in vitro. This interaction could potentially be used for targeting of NTHi with C4BP fusing proteins if the binding affinity can be increased by including different CCP domains and increasing the number of binding sites per molecule.

## Supplementary Material

Data Supplement
