# Supplementary material for: Nontypeable Haemophilus influenzae P5 Binds Human C4b-Binding Protein, Promoting Serum Resistance
Source: J Immunol. 2021 Sep 15;207(6):1566–77. doi: 10.4049/jimmunol.2100105 (PMC8428749; doi:10.4049/jimmunol.2100105)
Supplement: Data Supplement [file JI_2100105.zip › JI_2100105_Supplemental_1.pdf]

Supplementary Figures

```
                                                                   Loop 1
P5_3655            1 MKKTAIALVVAGLAAASVAQAAPQENTFYAGVKAGQGSFHDGINNNGAIK     50
                     |||||||||||||||||||||||||||||||||||||||||||||||.|.
P5_KR271           1 MKKTAIALVVAGLAAASVAQAAPQENTFYAGVKAGQGSFHDGINNNGVIG     50

                                                                      Loop 2
P5_3655           51 EALTSASYGYRRNTFTYGVFGGYQILNQDNFGLAAELGYDNFGRAKLREV    100
                     |||.|:.|||||||||||||||||||||||||||||||||:|||||:||.
P5_KR271          51 EALQSSGYGYRRNTFTYGVFGGYQILNQDNFGLAAELGYDDFGRAKIREA    100

                                                                 Loop 3
P5_3655          101 GKPSAKHTNHGAHLSLKGSYEVLDGLDVYGKAGVALVRSDYKRYEEANGT    150
                     |||.||||||||||||||||||||||||||||||||||||||.||.|||.
P5_KR271         101 GKPKAKHTNHGAHLSLKGSYEVLDGLDVYGKAGVALVRSDYKFYEVANGA    150

                                                                Loop 4
P5_3655          151 RNHDKGRHSLRTSGLFSVGAEYAVLPELAVRLEYQWLTRVGKLRTQDKPN    200
                     |:.::|||||||||||:|||||||||||||||||||||||||.|||||||
P5_KR271         151 RDRNQGRHSLRTSGLFAVGAEYAVLPELAVRLEYQWLTRVGKYRTQDKPN    200


P5_3655          201 SAINYNPWIGSINVGISYRFGQGEAPVVAAPEMVSKTFSLNSDVTFAFGK    250
                     ||||||||||||||||||||||||||||||||||||||||||||||||||
P5_KR271         201 SAINYNPWIGSINVGISYRFGQGEAPVVAAPEMVSKTFSLNSDVTFAFGK    250


P5_3655          251 ANLKPQAQATLDSVYGEISQVKSAKVAVAGYTDRIGSDAFNVKLSQERAD    300
                     ||||||||||||||||||||||||||||||||||||||||||||||||||
P5_KR271         251 ANLKPQAQATLDSVYGEISQVKSAKVAVAGYTDRIGSDAFNVKLSQERAD    300


P5_3655          301 SVANYFVAKGVAADAISATGYGEANPVTGATCDQVKGRKALIACLAPDRR    350
                     ||||||||||||||||||||||||||||||||||||||||||||||||||
P5_KR271         301 SVANYFVAKGVAADAISATGYGEANPVTGATCDQVKGRKALIACLAPDRR    350


P5_3655          351 VEIAVNGTK    359
                     |||||||||
P5_KR271         351 VEIAVNGTK    359

# Length: 359
# Identity:     341/359 (95.0%)
# Similarity:   348/359 (96.9%)
# Gaps:           0/359 ( 0.0%)
# Score: 1745.0
```

Supplementary Figure 1. Pairwise alignment of amino acid sequences of P5 from NTHi strains 3655 (P5_3655) and KR271 (P5_KR271). Global alignment was performed using EMBOSS Needle with the Needleman-Wunsch algorithm available at: https://www.ebi.ac.uk/).

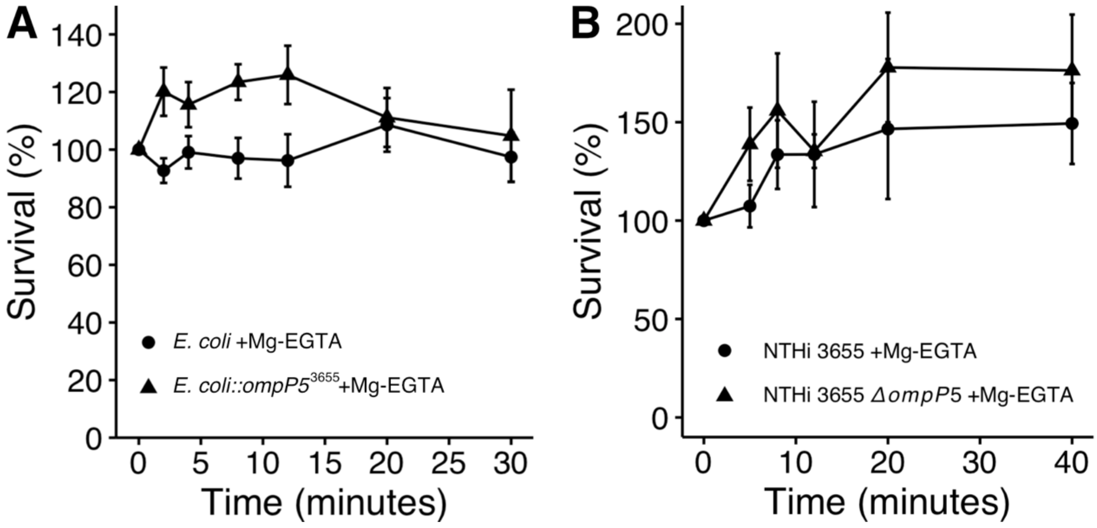

Supplementary Figure 2. (A) Serum resistance of *E. coli*, *E. coli*::*ompP5*^{3655} and *E. coli*::*ompP5*^{KR271} incubated with 0.5% NHS with Mg-EGTA (4 mM $MgCl_2$, 10 mM EGTA) at 37°C, assessed at different time points. **(B)** Serum resistance of NTHi strain 3655 and P5 mutant strain NTHi 3655*ΔompP5* incubated with 5% NHS with Mg-EGTA. Each bar represents the mean ± SEM of 3 to 8 independent experiments with technical duplicates.

Supplementary Tables

Supplementary Table I

Peptide name	Sequence	Number of residues
**NTHi 3655**		
P5_Loop1^{3655}	HDGINNNGAIKEALTSASYGYRRN	25
P5_Loop2^{3655}	GRAKLREVGKPSAKHTNHG	19
P5_Loop3^{3655}	SDYKRYEEANGTRNHDKGRHSLRTS	25
P5_Loop4^{3655}	TRVGKLRTQDKPNSAINYNPWIG	23
**NTHi KR271**		
P5_Loop1^{KR271}	HDGINNNGVIGEALQSSGYGYRRN	25
P5_Loop2^{KR271}	GRAKIREAGKPKAKHTNHG	19
P5_Loop3^{KR271}	SDYKFYEVANGARDRNQGRHSLRTS	25
P5_Loop4^{KR271}	TRVGKYRTQDKPNSAINYNPWIG	23

Primer	Sequence 5'-3'	Amplicon
**Construction of NTHi 3655*ΔompP5* and NTHi KR271*ΔompP5***		
CmR_For	ATGGAGAAAAAAATCACTGGA	Complete ORF of *cat* (69)
CmR_Rev	TTACGCCCCGCCCTGCCACTCAT	
UFP5_3655_F1[1]	TTTACGACACCTGCTTGTACTTTAACCCA	Upstream flanking region (UF-P5) of *p5* from NTHi 3655 with overlapping 5'sequence of *cat*
UFP5_3655-Cm_R1	TCCAGTGATTTTTTTCTCCATTTTGATGTCCTCTATTTAGTCATCGAAT	
DFP5_3655Cm_F1	ATGAGTGGCAGGGCGGGGCGTAATTTTAGTATTCGTTTAACGAAAGATT	Downstream flanking region (DF-P5) of *ompP5* from NTHi 3655 with overlapping 3'sequence of *cat*
DFP5_3655_R1[1]	CTGCCGTTGCGGTAATGAATGATGATGAA	
UFP5_271_F1[2]	TCATTCCGCCTAAAGTTGCTCGATTGCTGGTGGATAACTC	Upstream flanking region (UF-P5) of omp*P5* from NTHi KR271 with overlapping 5'sequence of *cat*
UFP5_271-Cm_R1	TCCAGTGATTTTTTTCTCCATTTTGATGTCCTCTATTTAGTCATCGAAT	
DFP5_271Cm_F1	TGAGTGGCAGGGCGGGGCGTAATTTTAGTCGTTTAACGAAAGATTAAATA	Downstream flanking region (DF-P5) of *p5* from NTHi KR271 with overlapping 3'sequence of *cat*
DFP5_271_R1[2]	ATTAACCGCTGCAGTTGCCGTAATGAATGACGATGAATTTATTGA	
**Cloning of full-length P5 into pET16b**		
FP5_3655_P16b[3]	CGGCCCCATGGGCATGAAAAAAACTGCAATCGCATTA	Full length P5 of NTHi 3655
RP5_3655_P16b[4]	CGTCCCCATATGTTATTTAGTACCGTTTACCGCGATT	
FP5_KR271_P16b[3]	GTGCCCCATGGGCAAAAAAACTGCAATCGCATTAGTAGT	Full length P5 of NTHi KR271
RP5_KR271_P16b[4]	GTCTCCATATGTTATTTAGTACCGTTTACTGCGATTTC	

[1, 2] Primer pairs were used in overlapping PCR to yield a linear *P5*-knockout cassette DNA as a mutation vector for knocking out *p5* in [a]NTHi 3655 and [b]NTHi KR271

[3, 4.] Restriction enzyme cutting sites are underlined. [c]*Nco*I, [d]*Nde*I

Supplementary References

69. Su, Y.-C., O. Mukherjee, B. Singh, O. Hallgren, G. Westergren-Thorsson, D. Hood, and K. Riesbeck. 2016. Haemophilus influenzae P4 Interacts With Extracellular Matrix Proteins Promoting Adhesion and Serum Resistance. *The Journal of infectious diseases* 213: 314-323.
